# Exploring Strategies for Using Social Media to Self-Manage Health Care When Living With and Beyond Breast Cancer: In-Depth Qualitative Study

**DOI:** 10.2196/16902

**Published:** 2020-05-25

**Authors:** Cathy Ure, Anna Mary Cooper-Ryan, Jenna Condie, Adam Galpin

**Affiliations:** 1 Directorate of Allied and Public Health School of Health and Society University of Salford Salford, Manchester United Kingdom; 2 School of Social Sciences and Psychology Western Sydney University Sydney Australia; 3 Directorate of Psychology and Sport School of Health and Society University of Salford Salford, Manchester United Kingdom

**Keywords:** breast cancer, social media, internet, self-management, psychosocial health, survivorship

## Abstract

**Background:**

As breast cancer survival rates improve and structural health resources are increasingly being stretched, health providers require people living with and beyond breast cancer (LwBBC) to self-manage aspects of their care.

**Objective:**

This study aimed to explore how women use and experience social media to self-manage their psychosocial needs and support self-management across the breast cancer continuum.

**Methods:**

The experiences of 21 women (age range 27-64 years) were explored using an in-depth qualitative approach. The women varied in the duration of their experiences of LwBBC, which facilitated insights into how they evolve and change their self-management strategies over time. Semistructured interviews were analyzed inductively using a thematic analysis, a polytextual analysis, and voice-centered relational methods.

**Results:**

The use of multiple social media platforms, such as YouTube, Facebook, WhatsApp, and Twitter, enabled women to self-manage aspects of their care by satisfying needs for timely, relevant, and appropriate support, by navigating identities disrupted by diagnosis and treatment and by allowing them to (re)gain a sense of control. Women described extending their everyday use of multiple platforms to self-manage their care. However, women experienced social media as both empowering and dislocating, as their engagement was impacted by their everyday experiences of LwBBC.

**Conclusions:**

Health care professionals (HCPs) need to be more aware, and open to the possibilities, of women using multiple social media resources as self-management tools. It is important for HCPs to initiate value-free discussions and create the space necessary for women to share how social media resources support a tailored and timely self-managed approach to their unique psychosocial needs.

## Introduction

### Background

Breast cancer remains the most common type of cancer in women [1]. Owing to the improvements in early diagnosis, treatment, and an aging population [2,3], survivorship rates and life expectancy for women living with and beyond breast cancer (LwBBC) are increasing. However, as health care systems are increasingly stretched, with significant gaps developing in health care resource provision, increasing patients’ abilities to engage in positive self-management behaviors when living with long-term conditions [4,5] has become a global issue [6].

Self-management—defined as “awareness and active participation by the person in their recovery, recuperation, and rehabilitation, to minimize the consequences of treatment, promote survival, health and well-being” [7]—is reported to support patient empowerment, increase self-efficacy, and lead to behavioral changes, while reducing demands on health care resources [4,5,7-9]. However, Rogers et al [9] suggest that more attention needs to be placed on the “contexts, resources, practices, priorities, and networks of patients living with a chronic condition to identify the nuanced ways in which self-care support and long-term condition management can be integrated into the open systems of people’s everyday lives.”

Women LwBBC report many ongoing and unmet psychosocial needs [10,11], including pain, fatigue, fear of recurrence, lymphedema, and hair loss [12-19]. As these complex issues require ongoing support, attending to the everyday resources, practices, priorities, and patient networks that women engage in for their own self-care, could offer health care professionals (HCPs) insights for better health care outcomes.

Social media have the potential to support women LwBBC to manage aspects of their own self-care, including managing unmet psychosocial needs. Social media are defined as a group of Web-based apps that enable the creation and sharing of user-generated content [20]. They offer patients instant access to information and new connections through easy access to other users [21]. Social media have become taken for granted health information resources [22,23] and include, for instance, a cancer tag ontology of hashtags on Twitter that links patients, doctors, caregivers, and advocates. This includes a weekly tweet chat in relation to breast cancer—#bcsm (breast cancer social media) [24,25]. However, research on social media use by women LwBBC is limited to a small number of studies [24,26-39]. These studies have focused either on discrete platform use (eg, Facebook, Twitter) or have adopted methods such as secondary data analysis [26-38], which do not acknowledge the complex ways in which social media are used and experienced by women LwBBC. Furthermore, research on social media as a component of electronic health interventions [6] ignores the experiences of women using commercial social media platforms to create, use, maintain, and generate grassroots informational and storytelling spaces, such as blogs, and community spaces, such as Facebook groups, to support their own self-management. Facebook groups can be *open*, *closed*, or *secret. Open* means anyone can see the group, who is in it, and can join it. *Closed* groups can also be seen by anyone. The group’s name, description, and member list are publicly visible; however, only those who have been invited to a closed group can see its posts. A *secret* group is not publicly visible. Women must ask to join secret breast cancer groups and are added by a moderator, and only group members can see group posts. As women are generating digitally mediated support together in Web spaces, understanding how women LwBBC use social media to support self-management and self-care practices can help target informational and psychosocial support more appropriately and provide useful information to HCPs about women’s self-management practices.

### Objective

This study aimed to explore how women use and experience social media to self-manage their psychosocial needs and support self-management across the breast cancer continuum.

## Methods

### Design

A qualitative study was used to explore women’s use of social media to support self-management when LwBBC as we were unable to find previous studies that explored use of multiple social media for this purpose. Semistructured interviews, including visual methods were developed to gain rich, detailed data [40]. Photographs were introduced into the interview setting through photo elicitation (social support images provided by the research team, eg, friends being together, families, HCPs, and mobile technologies, eg, women using mobile phones or laptops) or photo production (photographs taken by women to communicate their experiences of LwBBC) to elicit or trigger conversation [41]. An interview guide was developed by the research team.

### Sample and Sample Size

Women had to be 18 years or older, with a previous diagnosis of breast cancer. It was not a requirement that women had to use social media at the present time. We were interested in women’s experiences of social media use at any time since diagnosis. We were mindful that some women may have used social media at some point but had not found it helpful. It was as important to capture these experiences*.* We were interested in how women’s experiences of social media use varied in relation to time since diagnosis. We purposefully recruited women who had been LwBBC for less than twelve months, between 1 and 5 years, and 5 years or more, with equal numbers (n=7) recruited for each temporal period postdiagnosis. Participants emailed the lead researcher (CU) to register interest in participating after seeing study details online (in breast cancer Facebook groups [n=12], Twitter [n=2], charity websites [n=1]) or offline via cancer support centers (n=2), posters (n=1) or through word of mouth (n=3). In total, 44 women were interested in the study. Of the 44 women, 21 consented to participate, with 1 known to CU. Women decided which type of interview—photo elicitation or photo production—they wished to participate in.

### Data Collection

Face-to-face interviews carried out by CU were audio recorded and video recorded. The question structure was kept deliberately broad to enable women to have as much space as possible to explore their experiences of social media use. The first half of all interviews followed the same structure using the interview guide to ask broad questions related to personal experiences of breast cancer—women’s overall social media use and use in relation to LwBBC. In the interviews using photo-production techniques, women then shared the photographs they had taken to discuss how they communicated their experiences of LwBBC with others. In the interviews, using photo elicitation techniques, Wortman’s [42] study on social support was used to support probing questions. Photographs provided by the research team were used to prompt responses related to possible providers of social support, including HCPs, work colleagues, friends and neighbors, family including children and parents, partners, peers, and service providers, for example, charities. Interviews took place in university or community settings. Field notes were written after each interview. Considerations about data saturation were guided by the concept of information power [43], that is, the more relevant information a sample holds, relevant to the study, the fewer participants are needed—researcher subjectivity [44] and taking a pragmatic approach to sample size [45], given the resources available. On the consent form, women were asked how they wanted quotes to be credited, that is, with their own name or a pseudonym. Most participants (16/21, 76%) waived their rights to anonymity. Pseudonyms are used for those who retained anonymity. Ethical approval was granted by the University of Salford (approval number: HSCR 15-71).

### Data Analysis

**Figure 1:**
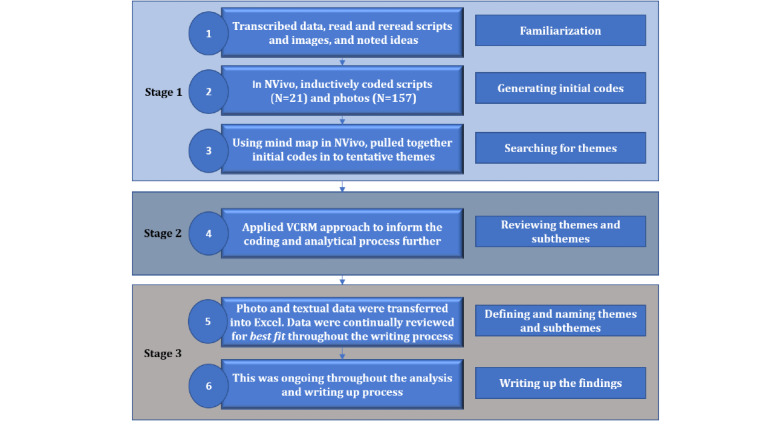
Data analysis process flowchart. (VCRM: voice-centered relational method).

Data were analyzed using thematic analysis [46], polytextual thematic analysis [47], and the voice-centered relational method [48] approaches. Figure 1 shows a review of the steps taken. Transcripts (including photographs) were inductively coded in NVivo (QSR International, version 11) by CU. Monthly review meetings were held with AMCR, AG, and JC to reflect on coding, to review field notes, and for thematic development.

### Participants’ Review

Participants were invited to review the findings via email. Of 21 participants, 18 (86%) responded to the invitation and were forwarded the findings. Three photo-elicitation participants did not respond to the emailed invitations. The summary of the findings invited participants’ responses by phone or email. Nine participants (9/18, 50%) responded. All (9) accepted the findings. Some offered comments about aspects of the findings that resonated with them. Others offered thanks for the opportunity to be involved.

## Results

### Characteristics of the Study Sample

A total of 21 women (age range: 27-64 years at the time of diagnosis) participated. Interviews lasted between 55 and 168 min (mean 99 min). The number of photographs (n=157) taken ranged from 3 to 47 (mean 17). Participants’ characteristics are provided in Table 1.

**Table 1:** Participants’ characteristics (N=21).

Characteristic		Values, n (%)
**Age at time of diagnosis (years)**		
	<31	3 (14)
	31-40	6 (29)
	41-50	9 (43)
	51-60	2 (9)
	>61	1 (5)
**Time since diagnosis**		
	<12 months	7 (33)
	1-5 years	7 (33)
	>5 years	7 (33)
**Marital status**		
	Single	5 (24)
	Cohabiting	2 (9)
	Married	13 (62)
	Divorced	1 (5)
**Ethnicity**		
	White British	19 (90)
	Black British	1 (5)
	Mixed or multiple ethnicities	1 (5)
**Employment status**		
	Full-time employment	7 (33)
	Part-time employment	4 (19)
	Unemployed	2 (9)
	Retired	3 (14)
	Student	2 (9)
	Not working through choice	1 (5)
	Unable to work due to health issues	5 (24)
**Number of times diagnosed**		
	Once	17 (81)
	Twice	4 (19)
**Type of breast cancer**		
	Primary	11 (52)
	DCIS^a^	4 (19)
	Primary and DCIS	3 (14)
	Local recurrence	2 (9)
	Secondary	1 (5)
**Treatments received**		
	Mastectomy	13 (62)
	Lumpectomy	11 (52)
	Chemotherapy	17 (81)
	Radiotherapy	14 (66)
	Tamoxifen	13 (62)
**Type of interview**		
	Photo elicitation	12 (57)
	Photo production	9 (43)
**Most popular social media platform use in relation to breast cancer**		
	Facebook	17 (81)
	YouTube	15 (71)
	WhatsApp	8 (38)
	Twitter	7 (33)

^a^DCIS: ductal carcinoma in situ.

### Themes

**Figure 2:**
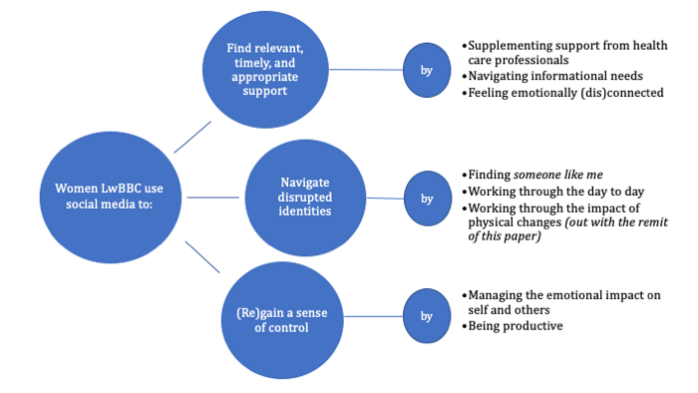
Thematic map: women's use of social media to support self-management when living with and beyond breast cancer.

A total of 3 main themes with 8 subthemes were identified in relation to why women use social media to support their experiences of LwBBC (Figure 2). For the purposes of this paper, the most relevant themes and subthemes that inform HCPs about women’s use related to self-care and self-management were discussed. Working through the impact of physical changes, a subtheme identified as part of the wider PhD study, was, therefore, not discussed here.

#### Theme 1: Finding Relevant, Timely, and Appropriate Support

Most women were active social media users at the time of their diagnosis. Extending their day-to-day use to find support was a logical extension of existing social media practices. Women described gaining support on social media through 3 subthemes: supplementing support from HCPs, navigating informational needs, and feeling emotionally (dis)connected.

##### Supplementing Support From Health Care Professionals

Women highly valued the clinical expertise provided by HCPs; however, collectively, they described themselves as cautious users of secondary health care provision. They avoided *mithering* or *bothering* breast care nurses, as *they never have enough time*. Phoning the breast care nurse was viewed as intrusive:

I don’t want to be bothering them with phone calls when they are in clinic.

Sarah, time since diagnosis <12 months

Therefore, women who described using social media to gain support felt unwilling or unable to access their HCPs. Indeed, all women using social media described how easy access to experiential support from other women LwBBC reduced their sense of needing to access HCPs. Furthermore, sometimes women decided that they no longer needed to seek reassurance from their general practitioner:

I mean I tried to make appointments with my GP, but you know the way things are going with the NHS [National Health Service] and all that. It is like three weeks until my next appointment. I don’t need to now. I just go on the group [Facebook group] and think’ oh, OK, alright yeah.

Jojo, time since diagnosis 1-5 years

Instead, women can experience closed and secret groups on Facebook, as providing immediate, relevant responses that reassure and inform:

There would be people to talk to rather than having to ring up the nurse, leave a message on the breast cancer nurse line, feeling really bad because they are really busy, and they are running around doing other things and then waiting for them to ring me back perhaps that same day perhaps not that same day erm, to this was...this immediacy that, you know, we get used to with technology erm...

Jayne, time since diagnosis 1-5 years

Women at all stages of the breast cancer continuum described their social media use as supplementing professional support and as a way to gain agency by removing their reliance on HCPs as the source of all breast cancer knowledge. By using social media to address questions and concerns quickly, women voiced notions of feeling empowered.

##### Navigating Own Informational Needs

Initially, women used information searching as a coping strategy. For some, information searching began after diagnostic testing and before formal diagnosis. Women reported experiencing information overload in the clinical setting and used social media to fill knowledge gaps at key points in their *patient journey*, outside of the formal clinical encounter:

Because there’s that much information, you can’t possibly take it all in at the appointment. And when they tell you, you are almost kind of shocked anyway. So, you don’t digest any of it. Nothing is retained. Absolutely nothing is retained.

Nicola, time since diagnosis 1-5 years

Women described *Googling* and seeking information from other women LwBBC on social media as common practice. Cancer charity websites, such as Cancer Research UK and Breast Cancer Care, were considered *legitimate*, *trustworthy*, and *up to date*. They were often women’s first port of call. However, when women wanted further information relating to their own specific experiences, they described these sites as providing insufficient breadth or depth to satisfy their needs. In addition, some women experienced informational support from breast cancer charities as leaflet driven, which was misaligned with their everyday information-seeking practices. Women supplemented static Web- and leaflet-based information with active (eg, liking, commenting, sending messages) and passive consumption (eg, lurking) [49,50] of other women’s experiences of LwBBC. Women described lurking in closed Facebook breast cancer groups immediately after diagnosis, which provided a *depth* of knowledge and built women’s confidence to advocate for themselves:

You kind of come across a post and there’ll be like 47 comments, by the time you’ve read all of that you’ve had quite an in-depth insight into that particular issue, so I just read a lot.

Kirsty, time since diagnosis <12 months

Using Twitter to follow and learn from other women, LwBBC was described as supporting joint decision making with HCPs:

I found Twitter really useful in that because then I started following lots of people, so by the time I spoke to people I was already pretty well informed, or I felt like I was anyway, um, obviously it gave me the opportunity to ask some questions then.

Sheena, time since diagnosis <12 months

In addition, women used YouTube to gain visual information relating to practical aspects of treatment and managing the effects of treatment, including lumpectomy, radiotherapy, and mastectomy procedures. Some women used YouTube to watch mastectomies *to see what they did* for greater knowledge and understanding.

Women supplemented Web-based information through active and passive consumption of experiential knowledge principally using Facebook, YouTube, and Twitter. By moving in and out of platforms and different groups on platforms, women gathered information at the appropriate time for them, determined by them. This supported women’s ability to cope with the amount of information they encountered when newly diagnosed and supported adjustment and informed anticipation of what the next stage in their breast cancer experience entailed at different stages of the breast cancer continuum. By engaging in seeking, sifting, evaluating, and sharing information, women validated their experiences and were better equipped to advocate for themselves across a range of everyday settings.

##### Feeling Emotionally (Dis)connected

Some women used different social media platforms to navigate feelings of disconnection from other people to mediate relationships that provided emotional support and to connect with other women who shared similar experiences. Many described family and friends as *uncomfortable* because *they don’t know what to say.* Some women talked about *my breast cancer* in everyday WhatsApp conversations. In this space, conversations about cancer were normalized, as women voiced their experiences while also providing family and friends with space and time to craft effective supportive responses. By publicly posting photographs as status updates on Facebook, women reported a sense that their story was being seen and heard. However, social media use was entangled in specific experiences. For instance, women showed highly tailored approaches to accessing emotional support at times, which were particularly challenging, such as the week of receiving chemotherapy. Women described these as important connections that reduced the sense of isolation and feelings of loneliness, which they felt unable to share with their family. Social media enabled connection with those who *understand* at a time when women felt disconnected from their normal support structures:

And those times when you are sat home for a week, bored out of your brains, feeling like death, it’s quite nice to connect with somebody that’s going through the same thing, yet you’ve not got the energy to talk so, you know what I mean, so it’s been really good for that because I think I would have felt quite lonely…yeah.

Sarah, time since diagnosis <12 months

This emotional connectedness was described by 2 women as *a lifesaver*:

I cannot think of a single source that would provide even close to the amount of...even close to the amount of support the YBCN [Younger Breast Cancer Network] has provided for me. I never looked elsewhere.

Delphi, time since diagnosis 1-5 years

Many women preferred gaining emotional support through closed or secret Facebook groups or other platforms such as WhatsApp, Skype, and FaceTime, as these digital spaces supported intimate conversations, feelings of proximity, and “the reality of it [breast cancer] sometimes.” Women described using different platforms simultaneously to scale how private or public they were about different aspects of their experiences. After finding social media groups or digital spaces that satisfied individual needs, some women developed personal relationships with other women LwBBC, which remained significant and important to them many years after their original diagnosis.

#### Theme 2: Navigating Disrupted Identities

Women described how breast cancer presented challenges to their sense of identity, which they navigated in numerous ways. The following 2 subthemes are relevant to HCPs: *finding someone like me* and *working through the day to day*.

##### Finding Someone Like Me

Postdiagnosis, many women used social media to find *someone like me*. Connecting with women in the *same boat*, who looked similar and were experiencing similar treatments helped reduce feelings of uncertainty. Women used different approaches to find women on social media, including looking for women of a similar age, with the same breast cancer type, and at the same stage *of the cancer journey.* Similarly, women targeted groups related to their experiences following particular treatments, including being *flat* postmastectomy, or having lymphedema. Most of the Facebook groups that women joined were grassroots, closed, and moderated:

A lot of us didn’t want reconstruction and some were thinking about going flat completely and one of them mentioned the Flat Friends group cos I, I wear a (pause) prosthesis; I didn’t have reconstruction I decided to join that group cos at one stage I thought erm do I go flat completely?

Millie, time since diagnosis >5 years

The need to find *similar others* on social media continued for some women along the breast cancer continuum. When women felt a difference between themselves and others, they sought out women in other groups they more closely identified with.

Some women identified difficulties with the notion of *someone like me* when they described experiencing the *hierarchy of suffering* [51], “whereby some kinds of suffering, pain, and misfortune are perceived as more difficult or signify a somewhat unique, superior source of suffering” (p.953). The *hierarchy of suffering* was described as invalidating personal experiences, and some women moved away from breast cancer discussions on Twitter and Facebook to actively manage their self-care. They described using social media to support their psychosocial health by extending friendships and interests with *similar others* with similar other interests, outside of breast cancer.

##### Working Through the Day to Day

Women at all stages of LwBBC described having to work through aspects of their breast cancer experience daily. For those with secondary breast cancer or further along the breast cancer continuum, many women detailed the impact and side effects of treatment as *constant reminders* and reported physical, emotional, psychological, and social challenges. Some women proactively used Twitter and Facebook to support themselves in the hospital setting while receiving treatment. This relieved pressure on the family to attend appointments, particularly for those living with secondary breast cancer:

I can’t have somebody coming every three weeks with me, it’s...who’s got time? Who’s got the energy? Who’s got the effort? I don’t mean that in a bad way, I know that it’s a drag.

Jo, time since diagnosis <5 years

For Jo, working through the day to day involved using social media to extend the clinical encounter to her social networks. She used her iPad to connect with her Twitter followers during treatment, drawing on support in real time as and when she needed it.

For some women, *work* was experienced by managing side effects daily as a result of their treatment. Social media, including YouTube, were used to find solutions to alleviate the discomfort experienced, including managing lymphedema. However, regular emotional labor was also required to cope with anxiety created by social media posts when women discussed nonadherence to clinical guidelines. A total of 11 women, all at different stages of the breast cancer continuum, discussed issues with tamoxifen adherence. Women shared how nonadherence and being challenged about their own adherence created dissonance and anxiety for others:

And then I look on the [...] network and quite a few people say, “why are you having Tamoxifen? I'd put up a fight against that. I'm not on Tamoxifen I don't think it's a good idea. I'm having this drug instead and erm” so again that [….] is, now I am having a bit of a worry and a bit of a wobble about being on this Tamoxifen.

Michelle, time since diagnosis <12 months

Although some women felt conflicted when other women LwBBC challenged clinical guidelines, many women used social media as a tool to gain a sense of control.

#### Theme 3: (Re)gaining a Sense of Control

Women described (re)gaining a sense of control through 2 subthemes: managing the emotional impact on self and others and being productive**.**

##### Managing the Emotional Impact on Oneself and Others

Women described seeking to control the emotional impact of LwBBC on others by shielding them from aspects of LwBBC. Often, women made use of messaging services when initially diagnosed to inform others to (re)gain some control over disseminating their *news*. In the closed Facebook groups, women at all stages of LwBBC were able to have conversations, which they felt they could not have, or did not want to have, with close family and friends. They described how conversations with other women LwBBC enabled them to be *more honest,* able to process concerns without putting additional *burden* on loved ones, and which they had control over in terms of timing:

through social media I think you can be a little bit more honest because (pause) you’ve not got as much invested in their feelings. If you know, what I mean and they’re going through it so you can’t shock or scare them or make them feel (pause). There’s no guilt in telling somebody on social media that yeah you do feel like shit, do you know what I mean, cos they’re not going to come rushing round to your house, so there’s that distance so I think you can definitely feel you can be more honest

Sarah, time since diagnosis <12 months

Women were purposeful in determining which platforms best supported their preferred communicative approaches and controlled when to publish personal information. WhatsApp provided women with a sense of intimacy, privacy, and connectedness, both with women LwBBC and their family/friends. Where women did not use WhatsApp and were not members of closed Facebook groups but were Facebook users, they posted to achieve responses that were (emotionally) manageable by controlling how they conveyed their experiences:

I would post “first out of six chemos. Last chemo – nailed it.” That kind of thing. Erm, I do put it as very matter of fact. I did not say anything like “chemo is crap. I feel awful.” And I would never post anything like erm, “I’m really down today” or anything like that. It was always very upbeat. I didn’t want anyone to pity me.

Wendy, time since diagnosis 1-5 years

Women also reported having to learn to protect themselves emotionally when using social media, as sometimes content was experienced as threatening. Women reported anxiety—“you don’t know what you’re going to find”—when searching for content or reading about others’ experiences. Women described strategies to control exposure to content so that it did not impinge negatively on their psychological health. This included prompt closing of content identified as having the *wrong atmosphere* and turning off push notifications from Facebook groups. Women described adopting flexible strategies of joining and leaving groups and conversations to reduce the negative impact on their sense of well-being across the breast cancer continuum. Any sense of information threat was met with a change in strategy, including avoidance and adaptation. Therefore, although women described social media as enabling them to compartmentalize and control aspects of their experiences, women also articulated the challenges of access to 24-hour information about breast cancer brings. Women emphasized the need to find ways to control access to other women’s experiences, continual contact with *support* groups, and different types of content.

##### Being Productive

Women LwBBC reported using their experiences productively through their social media use. In one way some women (re)gained a sense of control by creating contemporary social media–based health resources. These resources were often borne out of the lack of service provision and included the development of new Web-based spaces including Twitter chats (#bbcww) and Facebook groups: for younger women with breast cancer, to support children of parents affected by cancer, and for women who wish to remain *flat.*

Some women over 12 months postdiagnosis felt *productive* by *giving back* through sharing personal experiences with women more recently diagnosed. However, many acknowledged this support as draining and adapted their level of involvement and exposure to content to meet their own self-care needs at any given point in time.

Similarly, engaging with oncologists on social media was seen to support women in making decisions about their own health care. Examples of successful advocacy were acknowledged by others LwBBC as something to *learn from:*

I’ve been able to then go to my Oncologist and say, “Look at this, this is what they’re doing over in America, when are we getting it here?” Or “this is the treatment now available, when can I have it?”

Jo, time since diagnosis >5 years

For Jo, trying to influence other women LwBBC to develop positive attitudes toward improving their physical health provided purpose. By sharing updates on her own exercise goals, she sought to engage women in positive self-management behaviors. Jo used social media to inform, educate, and encourage others to be physically active to increase women’s chances of accessing future treatments or surgical procedures through a focused approach to healthy, active living.

Women’s photographs also demonstrated social media being used to actively challenge debilitating cancer narratives, which circulate in the mainstream press and on the Web. Countering problematic cancer narratives and having the right to reply was described as providing emotional release. Some women LwBBC, therefore, use social media as an opportunity for voice and reframing cancer conversations, which reduced their sense of disempowerment. Women can, therefore, develop complex social media identities that enable them to regain a sense of control through immersion in, shaping of, and sharing of expertise with others in ways that reciprocally supports their own individual needs.

## Discussion

### Principal Findings

This research identified 3 themes relating to women’s use of social media to support self-management: *finding timely, relevant, and appropriate support* (support); *navigating disrupted identities* (identity); and *(Re)gaining a sense of control* (control).

Women negotiate their entitlement to care [52] and use social media to supplement information provided by HCPs [53-55]. Significantly, women LwBBC at all stages of the breast cancer continuum are concerned about limiting demands on HCPs, for fear of *mithering* or *bothering.* This is supported by studies that show patients with cancer as reluctant to discuss psychosocial concerns with their clinicians [56] and users of online health communities (n=89) describing HCPs as *too busy for detailed discussions* [54]. To supplement the available support, some women are actively involved in digital labor in relation to meeting some of their psychosocial needs. Digital labor in a social media context relates to the unpaid creation of Web-based content and information [57]. In so doing, women articulate the perspective that they are reducing the potential demand for health resources. Rather than moving into new support environments postdiagnosis, such as using Web-based breast cancer charity forums, women’s digital labor includes adapting and changing the *social* spaces they already use or occupy on social media, as demonstrated with the collaborative establishment of #bcsm [24,25] on Twitter. Evidence suggests that those who have diverse networks—characterized by numerous and varied network members (family, friends, acquaintances, and groups) who are in frequent contact with the individual—have better self-management capabilities among those with long-term conditions [58]. Furthermore, those with diverse networks use formal health care services less often than those in restricted, minimal family, family, or weak tie networks, presumably because of the increased number of connections [58]. Most often, the social spaces used are a logical extension of everyday use with women moving across platforms to suit their unique needs [59], in line with the trend for users to have accounts or profiles on multiple platforms [60-62]. Facebook, YouTube, WhatsApp, and Twitter were the most frequently used platforms in this study. Women adopt complex Web-based searching strategies to satisfy multiple needs, including timeliness (immediacy), relatability (women like me), and authenticity (experiential experts).

The first theme reports how women, by moving in and out of different groups and social media platforms, gain a sense of self-efficacy by gaining information at the appropriate time for them, determined by them. This supports women’s ability to cope with the amount of information they encounter at challenging times along the breast cancer continuum and supports adjustment and informed anticipation of what the next stages in their cancer experiences entail. Using different social media platforms at different times demonstrates active and conscious decision making in tailoring connection and information seeking according to specific needs at any point in time.

Feeling an emotional connection to other women LwBBC was a significant factor in using social media, particularly when women felt disconnected from their usual support structures. Some women found that WhatsApp provided *mediated intimacy* [63] and supported the continuation of relationships postdiagnosis that felt disconnected or strained when face to face. By introducing everyday experiences of LwBBC into WhatsApp group conversations, women normalized conversations about cancer and succeeded in attending to their need to talk about their experiences. Women experienced WhatsApp as enabling message recipients to craft effective supportive responses.

The second theme captures the challenges for women in navigating disrupted identities and coping with *biographical disruption* [64-66]. Social media afford opportunities to link with and learn from *similar others*. When women experience a gap in service provision, they create or join niche groups [67] to find *likeminded* women. However, sometimes women find that participating in Facebook groups or on Twitter disrupts the validity of their experiences. They withdraw to better support their self-care. Women’s experiences encapsulate the ongoing debate about the *internet’s potential to create and diminish community* [68] with women experiencing participation as both positive and negative, sometimes simultaneously. More research is required to understand whether participation in social media conversations impacts adherence to clinical guidelines, including tamoxifen adherence and engagement in physical activities.

In the third theme—*(Re)gaining a sense of control*—women work to limit the *burden* of LwBBC on their families by self-managing aspects of emotional work through other women on social media. WhatsApp and Facebook Messenger were sometimes used following initial diagnosis to inform others. This was protective of psychosocial health by removing uncertainty around sharing the *diagnostic narrative* and afforded a sense of control through framing experience to influence the type of response required. On Twitter and in closed and secret groups, managing exposure to other women’s experiences is challenging. Strategies employed to limit exposure to potentially threatening content support previous research [69] and align with women’s approaches to information searching in relation to breast screening [70]. Similarly, push notifications on smartphones controlling the flow of information [71] from breast cancer groups acted as negative *constant reminders* of breast cancer. Being able to control information flow by changing notifications enabled a sense of control. The ability to use social media to compartmentalize the experiences of LwBBC was seen as a benefit of social media use.

### Clinical Implications

Although evidence suggests that social media are shifting aspects of the patient-provider relationship [72], the original research aims of this study did not include generating outcomes for HCPs as a specific objective. However, the findings indicate that there are opportunities for HCPs and patients to work closer together to understand the benefits of social media use to support self-management. To encourage open conversations with patients, it may be useful for HCPs to encourage women LwBBC to share if and how they are addressing their self-management needs through their interactions with other women on social media. Given that many breast cancer groups on Facebook are closed or secret and WhatsApp groups are encrypted, there are challenges for researchers and HCPs alike in gaining awareness of what is shared in these spaces without dialog. Understanding which platforms or specific groups women find useful as self-management tools could enable practitioners to signpost other women to social media resources that women find beneficial. Discussing the knowledge gained by women through platforms and social media groups provides opportunities for HCPs to support women’s appraisal of information and could encourage open discussion about different approaches to self-management. Furthermore, there are opportunities for breast care nurses and oncologists to guest-moderate *chats* in grassroots social media spaces to support women’s decision making and strategies for self-management.

### Limitations

This was a qualitative study to understand the complexity of women’s social media use when LwBBC. However, it did not provide insight into the relative extent of different aspects of social media use across a broad and representative population and did not provide insights into men’s experiences of social media use when LwBBC. Further research should attempt to capture quantitative data to identify how social media use develops self-efficacy when LwBBC, supports self-management behaviors and impacts the overall sense of health and well-being across the cancer continuum.

### Implications for Future Research

The ability to determine how, when, and where to access 24-hour support using social media provides opportunities for women globally to proactively engage in self-management practices unavailable a decade ago. Although women use social media in part to reduce demand on health care services, it is unknown whether use supports decision making or exacerbates issues within the clinical setting, for example, through poor decision making, which later increases demand for clinical services. Understanding HCPs’ perceptions of the use of social media to support 24-hour self-managed care is an area for further research inquiry.

## Abbreviations

bcsm: breast cancer social media
HCP: health care professional
LwBBC: living with and beyond breast cancer
